# Awareness of Obstetric and Delivery Complications Among Saudi Pregnant Women in Riyadh, Saudi Arabia

**DOI:** 10.7759/cureus.39630

**Published:** 2023-05-29

**Authors:** Joud Sami M Maki, Hattan Dagestani, Laila Aldokhail, Lujain Mohamed Alaradi, Mohammed Albalawi

**Affiliations:** 1 College of Medicine, King Saud bin Abdul-Aziz University for Health Sciences, Riyadh, SAU; 2 Obstetrics and Gynecology, King Abdul-Aziz Medical City, Ministry of National Guard-Health Affairs (MNG-HA), Riyadh, SAU; 3 College of Medicine, Princess Nourah bint Abdulrahman University (PNU), Riyadh, SAU; 4 College of Medicine and Medical Sciences, Arabia Gulf University, Manama, BHR

**Keywords:** pregnancy, complications, delivery, obstetric, awareness

## Abstract

Introduction: The birth of a newborn is often celebrated with delight and excitement around the world. However, maternal mortality remains a great concern, and the majority of these deaths could have been prevented. This study aims to assess the awareness of obstetric and delivery complications among pregnant women in Riyadh, Saudi Arabia.

Methodology: A cross-sectional study was conducted among 385 pregnant women attending antenatal care clinics in Riyadh. The participants were interviewed using a pre-tested questionnaire that included sociodemographic and obstetric data, as well as 16 questions to measure awareness of danger signs during pregnancy, labor, and the postpartum period, and knowledge of Birth Preparedness and Complication Readiness (BPCR).

Results: Among the 385 pregnant women, only 45.5% were aware of associated complications during pregnancy, 18.4% during labor, and 30.6% during the postpartum period. Although 82% of the women had heard about BPCR previously, only 53% took action toward it. Certain factors, such as age, level of education, having a medical condition, and the number of antenatal care clinic visits, were associated with an increased level of awareness.

Conclusion: The study highlights a lack of awareness regarding obstetric and delivery complications among Saudi pregnant women. Therefore, dedicated education by a healthcare provider during prenatal visits is recommended to increase knowledge and avoid future obstetric complications.

## Introduction

The birth of a newborn is celebrated with delight, excitement, and fulfillment in most cultures globally. However, at any point during antepartum, intrapartum, and postpartum, unexpected, or life-threatening events can occur. According to the World Health Organization (WHO), approximately 295,000 women died during pregnancy, delivery, and postpartum period in 2017. The most frequent causes of maternal deaths include hemorrhage, sepsis, hypertensive disorders, unsafe abortion, infection, obstructed labor, and complications during delivery [1,2]. Life-threatening complications are often preventable and treatable if proactive and prompt interventions are administered, including primary preventions such as raising good awareness of the obstetric signs, and complications and preparing for birth. Implementing such interventions will support the timely use of skilled maternal and neonatal care, particularly during childbirth. Evidence indicates that Birth Preparedness and Complication Readiness (BPCR) interventions can reduce maternal and neonatal mortality, which includes five components: finding a qualified birth attendant, identifying a healthcare facility, arranging the transportation, identifying a blood donor, and financial preparedness for emergencies and being knowledgeable about the danger sign [3-5].

In many studies, mothers were evaluated on whether they have the required awareness of BPCR by measuring the level of preparedness which includes the recommended five measures while pregnant: finding a qualified birth attendant, identifying a health care facility, transportation arrangements, identifying a blood donor, and prepared money for emergencies and whether they know the danger signs that might occur during pregnancy, delivery and after. Being well-prepared meant taking at least three steps and knowing at least two danger signs [3-6]. Most of the studies suggested that raising awareness in early adolescence and improving healthcare programs were the best way to improve BPCR and avoid future complications [7-14]. Thereupon, the choice of this topic was based on a previous observational study, highlighting the lack of preparedness among Saudi women in terms of obstetric and delivery complications [15]. Based on prior evidence and a deficit of local studies, this study aims to assess the level of awareness of obstetric and delivery complications among pregnant Saudi women in Riyadh, Saudi Arabia, and to identify the factors associated with the level of awareness to prevent future obstetric complications.

## Materials and methods

A community-based, cross-sectional survey was conducted at the antenatal clinics (ANC) in three different governmental hospitals (King Abdullah bin Abdulaziz university hospital, national guard hospital, and prince sultan military medical hospital) in Riyadh City, the capital city of Saudi Arabia. Via random sampling technique, participants in the waiting area of the ANC in the three different hospitals were selected and interviewed. The study included all Saudi pregnant females either primigravida females or multigravida females who were above 18 years old from various sociodemographic and socioeconomic statuses. Women who were physically or mentally retarded and those who refused to be interviewed were excluded from the study. Selected participants were then interviewed by the research assistants using a self-developed questionnaire, adapted from John Hopkins Program for International Education in Gynecology and Obstetrics (JHPIEGO) [3]. In addition, a pilot study was done to ensure the questionnaire's validity and resolve any ambiguities.

The questionnaire comprised socio-demographic variables including age and educational status, obstetric variables, gestational age of the current pregnancy, parity, medical conditions, and the number of antenatal visits. Moreover, after copyright was obtained for using the questionnaire there were 16 focused questions to measure the awareness of BPCR, which includes the five main components (finding a qualified birth attendant, identifying a healthcare facility, transportation arrangements, identifying a blood donor, and preparing money for emergencies), being well-prepared meant taking at least three steps and knowing at least two danger signs and the complications during pregnancy, labor/childbirth, and the postpartum period. To assess the level of awareness, a scoring system, adapted from JHPIEGO, was used in which participants were asked to list at least three of the 12 major danger signs during pregnancy, which include severe vaginal bleeding, swollen hands or face, and blurred vision, spontaneously to be considered fully aware. Similarly, full awareness of labor/childbirth complications was defined by spontaneously mentioning at least three of the nine major danger signs during labor, which include severe vaginal bleeding, prolonged labor (labor lasting for more than 12 h), convulsions, and retained placenta. In terms of postpartum period complications, full awareness of postpartum complications was defined as mentioning spontaneously at least three of the 11 key danger signs, which include severe vaginal bleeding, foul-smelling vaginal discharge, and high temperature. Finally, the test was considered significant if the p-value was less than 0.05. Informed consent was obtained from each participant before the interview, and the study was conducted after obtaining ethical approval from the Institution Review Board (IRB) at King Abdullah International Medical Research Center, protocol number RSS22R/020/07, starting from August 2022 to November 2022. The sample size was calculated based on an infinite population and estimated as 50%. Using a 95% confidence interval and a 5% margin of error, the calculated sample size was 385, adjusted to 400, to compensate for incomplete questionnaires. The sample size was calculated using a Raosoft calculator. The data were entered in a customized data collection Excel sheet. The data were reviewed and entered in the statistical package and analyzed using SPSS v.26.0 (IBM Corp., Armonk, NY). The data collection sheet contained descriptive statistics which included categorical data such as frequency and percentage. Chi-square test, t-test, and ANOVA test were used to assess the association between the demographic factors and the level of knowledge.

## Results

In this study, out of 400 participants, 385 participants were eligible and candidates for the interview. Data were collected from 385 pregnant women in the Riyadh region, Saudi Arabia. Of the participants, the participants' (53.0%) age range was 25 to 34 years, and 31.4% were older than 35 years old. The majority (87.8%) had a secondary school or higher as the highest educational level. Less than three-quarters of the participants' (70.6%) gestational age was more than six months, and 79% had between four and six living births. A small proportion (20.3%) of the women reported medical conditions, and the majority (94.5%) attended the ANC regularly (Table 1).

**Table 1:** Demographic and obstetrics characteristics *Antenatal care (ANC)

		n	%
Age (years)	15-24	48	12.5
	25-34	204	53.0
	35-45	121	31.4
	Above 45	12	3.1
Education status	Illiterate	4	1.0
	Elementary school	8	2.1
	Middle school	35	9.1
	Secondary school or above	338	87.8
Gestational age (months)	1-3	41	10.6
	4-6	72	18.7
	More than 6	272	70.6
Parity	0	2	0.5
	1-3	79	20.5
	4-6	304	79.0
Medical conditions	No	307	79.7
	Yes	78	20.3
*attending the minimum recommended number of ANC visits	No	355	92.2
	Yes	30	7.8

In terms of BPCR, 82.6% reported that they had heard about BPCR, and more than half (53.5%) of participants identified that the Internet was the main source of information regarding BPCR, and only 38.4% of the participants received education in the clinic regarding BPCR. Moreover, a small proportion of the participants (7.8%) participated in community activities related to birth preparedness such as workshops and courses. Although 82.6% of the participants reported that they had heard about BPCR, only more than half (53.8%) of the participants reported that they took action to prepare for birth by arranging a blood donor, transportation, place for birth and taking courses. barrier for the mother to give birth at a health facility. In addition, 54.5% reported that their community provides services to assist women in preparing for birth which includes transportation, ways to get a blood donation, and courses, with 23.9% not knowing if such services are provided. Almost a third of the participants (29.4%) reported that lack of transport as the main for the mother to give birth at a health facility (Table 2). Considering the awareness of the complications occurring during the different stages of pregnancy, bleeding was the most known complication (71.3%), followed by water break (30.0%) and abdominal pain (29.2%). In terms of childbirth-associated complications, severe bleeding (58.7%), prolonged labor of more than 12 hours (23.4%), and preeclampsia (19.8%) were the most reported complication. Regarding postpartum complications, bleeding (50.1%) was the top-mentioned complication, followed by depression (39.4%) and fever (20.6%) (Table 3). Although a high number 62.9% of cases knew that pregnant women could die from any of these problems, and 84.9% knew that maternal complications could endanger the life of the baby, almost half of the participants were partially aware of the associated complications in the following phases: during pregnancy (47.2%), childbirth (41.8%), and postpartum (49.8%), respectively. Moreover, less than half of the participants were fully aware of the dangerous obstetric signs. For the complications during pregnancy, only 45.1% were fully aware of the complications. In terms of childbirth complications, 38.9% were fully aware. Regarding the postpartum period complications, only a third of the participants (30.3%) were fully aware of the complications in the postnatal period (Table 4). 

**Table 2:** Birth preparedness and complication readiness (BPCR)

		n	%
exposure to BPCR	No	67	17.4
	Yes	318	82.6
Source of BPCR	Healthcare professional	45	11.7
	Brochures	5	1.3
	Internet	206	53.5
	Book	18	4.7
	Other	49	12.7
	None	62	16.1
Actions related to BPCR	No	178	46.2
	Yes	207	53.8
Availability of BPCR services	No	83	21.6
	Yes	210	54.5
	I do not know	92	23.9
Importance of transportation in BPCR	No	132	34.3
	Yes	113	29.4
	I do not know	140	36.4
Exposure to BPCR in ANC	No	237	61.6
	Yes	148	38.4
Participation in BPCR activities	No	355	92.2
	Yes	30	7.8

**Table 3:** Proportion of women who reported knowledge of key danger signs during pregnancy, childbirth, and postpartum

Knowledge of danger signs in pregnancy	N	%
Bleeding	273	70.0
Water breaks without labor.	115	29.8
Severe abdominal pain	112	29
Preeclampsia	101	26
Accelerated/reduced fetal movement	86	22.3
Severe headache	60	15.5
High fever	51	13.2
Swollen hands/face and feet	48	12.4
Difficulty breathing	45	11.6
Severe weakness	34	8.8
Loss of consciousness	33	8.5
Convulsions	19	4.9
Knowledge of danger signs in childbirth	N	%
Severe bleeding	178	58.5
Labor lasting > 12 hours	71	23
Preeclampsia	61	20
Perineal tear	47	15.4
High fever	31	10%
Placenta was not delivered 30 minutes after the baby was delivered	31	10
Severe headache	22	7.2
Loss of consciousness	22	7.2
Convulsions	13	4.2
Complications during the period postpartum period	N	%
Severe bleeding	192	49.8
Depression	152	39.4
High fever	79	20.5
Perineal tear	61	15.8
Severe weakness	40	10.3
Blurred vision	36	9.3
Difficulty breathing	36	9.3
Loss of consciousness	34	8.8
Severe headache	30	7.7
Swollen hands/face and feet	25	6.4
Convulsions	17	4.4

**Table 4:** Knowledge of obstetric and delivery complications

Complications during pregnancy	N	%
Not aware	29	7.5
Partially aware	182	47.2
Fully aware	174	45.1
Complications during Childbirth	N	%
Not aware	74	19.2
Partially aware	161	41.8
Fully aware	150	38.9
Complications during the period after childbirth	N	%
Not aware	76	19.7
Partially aware	192	49.8
Fully aware	117	30.3

Furthermore, certain factors were found to be related to the level of awareness. A significant positive correlation exists between age and knowledge of antepartum, intrapartum, and postpartum period complications (P=0.029, 0.042, and 0.007) (r=0.139, 0.105, and 0.146) indicating that older females have a better level of knowledge compared to younger participants. Another significant positive correlation was found between the educational level of the participants (P=0.041); however, the educational level is only significant in the awareness of the danger signs during pregnancy complications, but no significant association was found between the level of education and the level of knowledge regarding complications associated with labor and in the postnatal period (r=0.22, 0.061, P=0.571, 0.601). Parity was not associated with any of the knowledge categories (P=0.068, 0.690,0.202). A known history of medical condition was significantly correlated with better awareness of the danger signs during pregnancy and postpartum periods (r=0.150, 0.155, P=0.003, 0.002), yet it was not associated with awareness of childbirth complications (r=0.01, P=0.841) (Table 5).

**Table 5:** Factors associated with increased awareness level about antepartum, intrapartum, and postpartum complications * Significant when the P-value is lower than 0.05 *ANC = Antenatal Care

		Awareness of complications during pregnancy			Awareness of complications during Labor			Awareness of complications in postnatal period			
		Not aware N (%)	Fully aware (N (%)	P-value	Not aware N (%)	Fully aware (N (%)	P-value	Not aware N (%)	Fully aware (N (%)	P-value	
Age	15-24	33 (15.7)	15 (8.6)	0.029*	45 (14.3)	3 (4.2)	0.042*	40 (15.0)	8 (6.8)	0.007*	
	25-34	113 (53.8)	91 (52.0)		166 (52.9)	38 (53.5)		142 (53.2)	62 (52.5)		
	35-45	61 (29.0)	60 (34.3)		93 (29.6)	28 (39.4)		81 (30.3)	40 (33.9)		
	Above 45	3 (1.4)	9 (5.1)		10 (3.2)	2 (2.8)		4 (1.5)	8 (6.8)		
Education status	Illiterate	2 (1.0)	2 (1.1)	0.041*	3 (1.0)	1 (1.4)	0.575	3 (1.1)	1 (0.8)	0.601	
	Elementary school	5 (2.4)	3 (1.7)		8 (2.5)	0 (0.0)		7 (2.6)	1 (0.8)		
	Middle school	27 (12.9)	8 (4.6)		28 (8.9)	7 (9.9)		26 (9.7)	9 (7.6)		
	Secondary school or above	176 (83.8)	162 (92.6)		275 (87.6)	63 (88.7)		231 (86.5)	107 (90.7)		
Parity	0	2 (1.0)	0 (0.0)	0.086	2 (0.6)	0 (0.0)	0.690	2 (0.70)	0 (0.0)	0.220	
	1-3	50 (23.8)	29 (16.6)		66 (21.0)	13 (18.3)		60 (22.5)	19 (16.1)		
	4-6	158 (75.2)	146(83.4)		246 (78.3)	58 (81.7)		205 (76.8)	99 (83.9)		
Medical condition	No	179 (85.2)	128 (73.1)	0.003*	251 (79.9)	56 (78.9)	0.84	224 (83.9)	83 (70.3)	0.002*	
	Yes	31 (14.8)	47 (26.9)		63 (20.1)	15 (21.1)		43 (16.1)	35 (29.7)		
ANC visits	No	16 (7.6)	5 (2.9)	0.040*	19 (6.1)	2 (2.8)	0.278	18 (6.7)	3 (2.5)	0.094	
	Yes	194 (92.4)	170 (97.1)		295 (93.9)	69 (97.2)		249 (93.3)	115 (97.5)		
Exposure to Birth Preparedness and complication readiness (BPCR)	No	48 (22.9)	19 (10.9)	0.002*	63 (20.1)	4 (5.6)	0.004*	53 (19.9)	14 (11.9)	0.057	
	Yes	162 (77.1)	156 (89.1)		251 (79.9)	67 (94.4)		214 (80.1)	104 (88.1)		

## Discussion

The birth of a is celebrated with delight, excitement, and fulfillment in most cultures globally. However, at anyone during pregnancy, childbirth, and after childbirth, a life-threatening event can occur. According to the World Health Organization (WHO), three delays are associated with maternal mortality across countries, namely a delay in deciding to seek care, a delay in reaching a place of care, as well as a delay in receiving appropriate and adequate care [16]. Thereupon, adequate awareness by females of the danger signs of obstetric complications is the first step in preventing such events. The finding of this study highlights the lack of awareness of the danger signs during the antepartum, Intrapartum, and postpartum periods, and only half of the participants took action toward BPCR. Furthermore, the results of the study demonstrate that certain factors such as age, educational level, known his- tory of a medical condition, parity, and the number of Antenatal Organization (WHO), three delays are associated with maternal mortality across countries, namely a delay in deciding to seek care, a delay in reaching a place of care, as well as a delay in receiving appropriate and adequate care [16]. Thereupon, adequate awareness by females of the danger signs of obstetric complications is the first step in preventing such events.

The finding of this study highlights the lack of awareness of the danger signs during the antepartum, Intrapartum, and postpartum periods, and only half of the participants took action toward BPCR. Furthermore, the results of the study demonstrate that certain factors such as age, educational level, known history of a medical condition, parity, and the number of ANC visits are associated with the level of awareness. First, BPCR is defined as the process of planning and managing for normal delivery and anticipating the required actions that are needed to deal with an emergency. Interestingly, the result of the current study shows that 82.6% of pregnant Saudi women were exposed previously to BPCR, which is slightly higher than reported by Teekhasaenee, Kaewkiattikun in which it was reported that 78.4% of the women knew about BPCR [11]. Furthermore, another study, which was conducted in Tanzania, concluded that only 59% of the participants were aware of the five key components of BPCR [17]. The differences in percentages between the current study’s findings and the other studies’ findings can be explained by two main factors. First, the participants' educational level, as it was reported by Bintabara et al., that 66.8% of the study's subjects had attained primary and secondary education [17]. Another key factor is the participants' age range, as in Teekhasaenee, Kaewkiattikun, in which it was reported that 78.4% of the women knew about BPCR, the participants' age ranged between 10 and 19 years old aiming to assess pregnant adolescents' awareness toward BPCR. Despite the current study's finding, a high number of cases knowing about BPCR, only 53.8% of the participants took action to prepare for birth by arranging a blood donor, transportation, and birthplace, and acquiring adequate learn by attending workshops. This finding can be explained by the free and easy access to competent hospitals all over the region.

In addition, in this study, less than half of the women were aware of the possible complications that could occur during pregnancy followed by only 18.4% and 30.6% of women who were fully aware of complications during labor and in the postnatal period. The reported finding is higher than the reported finding by Abu-Shaheen et al., in which it was reported only 13.5% of the women were knowledgeable about the three complications during pregnancy followed by 3.4% who were able to identify three complications during labor and the postpartum period [15]. This finding might indicate inadequate education by the healthcare provider during the antenatal visit as the study shows that only 38.4% of the participants received enough education in the clinic. On the contrary, more than half (53.5%) of participants identified that the Internet and social media might provide inaccurate information. Therefore, Teaching and emphasizing the danger signs should be included in the first health education program during the antenatal period. However, knowing the danger signs would not necessarily ensure that pregnant women could recognize the severity of the problems. Healthcare personnel should also inform all pregnant women to be aware of the danger signs as well as go to the hospital as soon as such complications occur. Most of the participants knew that bleeding is a complication during pregnancy, labor, and the postpartum period. They also identified breaking the water and abdominal pain during pregnancy, prolonged labor of more than 12 h, and preeclampsia during labor, as well as depression and fever during the postpartum period as danger signs. The proportion who was aware that bleeding is a danger sign was higher than reported in several studies [18-20]. However, regarding the danger signs during labor, a higher percentage of women knew prolonged labor as a danger sign compared to the literature [12,21].

the current study's findings suggest that certain factors such as age, having a medical condition, parity, number of ANC visits, and adequate knowledge about BPCR, were significantly associated with the level of knowledge toward the danger signs during pregnancy, labor and the postpartum period. In regard to age, females above 25 years of age had better awareness of obstetric complications than the younger age group. This finding is consistent with Kabakyenga et al., in which it was reported that females older than 25 years had better awareness. This finding can be explained by two key factors. First, the participants educational level, as 87% of the current study's participants had attained secondary education or above. Second, the majority (79%) of the study's subjects were multigravida which was shown to be positively correlated to the level of awareness according to Bayu et al. [22]. Another factor that was shown to be correlated to a better level of awareness is a longstanding history of a medical condition as the current study finding demonstrates that having a medical condition is associated with better awareness regarding the danger signs during the antepartum and postpartum period, but is not associated with the intrapartum period, which can be explained by the fact that females with maternal morbidity are more likely to undergo a cesarean section, as a consequence, leading to deficient knowledge toward normal delivery and its associated complications [23,24]. A surprising finding in the study is that parity, the number of living births was not associated with better knowledge regarding the danger of obstetric signs. On the other hand, several studies suggested the synergistic effect of parity on the awareness of danger signs during pregnancy, childbirth, and after childbirth [16,21]. Additionally, the number of ANC visits was significantly associated with better knowledge of the danger signs during pregnancy, labor, and the postpartum period. This is similar to the results in the literature indicating that the number of ANC visits was associated with better knowledge [11,25-28]. The limitations of the study, interviewer bias is a possible limitation as the study was self-reported. Although a sample size consisting of 400 participants is sufficient for a community-based study, a nationwide study with a larger sample size is recommended for better representation.

## Conclusions

In conclusion, the majority of women had heard about BPCR; however, participants demonstrated low awareness of obstetric and delivery complications. The study also demonstrates a significant association between age, educational level, known history of a medical condition, and frequent ANC visits with an increased level of awareness. This warrants that awareness programs during the ANC visits and public service advertising campaigns would be effective at reaching individuals and raising awareness to prevent future obstetric and delivery complications.
